# Dopaminergic Suppression of Synaptic Transmission in the Lateral Entorhinal Cortex

**DOI:** 10.1155/2008/203514

**Published:** 2008-08-25

**Authors:** Douglas A. Caruana, C. Andrew Chapman

**Affiliations:** Center for Studies in Behavioral Neurobiology, Department of Psychology, Concordia University, Montréal, Québec, Canada H4B 1R6

## Abstract

Dopaminergic projections to the superficial layers of the lateral entorhinal cortex can modulate the strength of olfactory inputs to the region. We have found that low concentrations of dopamine facilitate field EPSPs in the entorhinal cortex, and that higher concentrations of dopamine suppress synaptic responses. Here, we have used whole-cell current clamp recordings from layer II neurons to determine the mechanisms of the suppression. Dopamine (10 to 50 *μ*M) hyperpolarized membrane potential and reversibly suppressed the amplitude of EPSPs evoked by layer I stimulation. Both AMPA- and NMDA-mediated components were suppressed, and paired-pulse facilitation was also enhanced indicating that the suppression is mediated largely by reduced glutamate release. Blockade of D_2_-like receptors greatly reduced the suppression of EPSPs. Dopamine also lowered input resistance, and reduced the number of action potentials evoked by depolarizing current steps. The drop in input resistance was mediated by activation of D_1_-like receptors, and was prevented by blocking K^+^ channels with TEA. The dopaminergic suppression of synaptic transmission is therefore mediated by a D_2_ receptor-dependent reduction in transmitter release, and a D_1_ receptor-dependent increase in a K^+^ conductance. This suppression of EPSPs may dampen the strength of sensory inputs during periods of elevated mesocortical dopamine activity.

## 1. INTRODUCTION

The entorhinal cortex is an important interface that links primary sensory and association cortices to the hippocampal formation, and it is critical for the sensory and mnemonic functions of the medial temporal lobe [1–4]. In the rat, the lateral division of the entorhinal cortex receives most of its cortical inputs from the olfactory cortex and perirhinal cortex, and the medial entorhinal cortex receives visual and multimodal inputs mainly via the postrhinal cortex [5–7]. This pattern of cortical input to the medial and lateral divisions of the entorhinal cortex contributes to their different roles in sensory and cognitive processing [8–10]. In addition, neuromodulatory transmitters innervate both the medial and lateral entorhinal cortices and can have powerful effects on sensory and mnemonic function in these regions. Specifically, acetylcholine and serotonin both modulate synaptic transmission and rhythmic EEG activities in the medial entorhinal cortex [11–15]. Further, midbrain dopamine neurons send one of their largest cortical projections to the superficial layers of the lateral entorhinal cortex where they target principal cell islands [16–18]. Relatively little is known, however, regarding the neuromodulatory effects of dopamine in the lateral entorhinal cortex.

The large dopaminergic projection to the prefrontal cortex is known to regulate cellular processes related to working memory [19–21], and dopaminergic inputs to the lateral entorhinal cortex are also likely to affect mechanisms of sensory and mnemonic function. In the prefrontal cortex, activation of D_1_ receptors can suppress glutamate release in layer V [22–24] but can enhance glutamatergic transmission in layer III [25, 26]. Further, the positive effect of D_1_ receptor activation on working memory follows an inverted U-shaped function [27], and strong or weak stimulation of D_1_ receptors can also have opposite effects on NMDA receptor-mediated synaptic currents [20, 28]. We have also found that dopamine has dose-dependent bidirectional effects in layer II of the lateral entorhinal cortex. In awake animals, increasing levels of dopamine with a selective reuptake inhibitor facilitates synaptic responses evoked by stimulation of the piriform cortex, and field excitatory postsynaptic potentials (fEPSPs) are also facilitated by a low concentration of dopamine in vitro [29]. Higher concentrations of dopamine, however, suppress fEPSPs, and similar suppression effects have been observed by others in medial entorhinal cortex layer II [30] and layer III [31]. Dopamine can also reduce the input resistance of layer II neurons in the medial entorhinal cortex [30] and reduce temporal summation in layer V neurons of the lateral division through an increase in the *I*_h_ current [32]. Dopamine may therefore modulate synaptic function in the lateral entorhinal cortex through multiple mechanisms.

 We have used whole-cell current clamp recordings to investigate the mechanisms of the suppression of EPSPs by dopamine in electrophysiologically identified “fan” cells in layer II of the lateral entorhinal cortex. Receptor blockers were used to determine the dopamine receptors that mediate the suppression of EPSPs, and paired-pulse tests were used to assess whether the suppression is expressed pre- or postsynaptically. Changes in the intrinsic excitability of fan cells were also monitored using responses to hyperpolarizing and depolarizing current steps. In addition to a D_2_-like receptor-mediated suppression of transmitter release, we show evidence that EPSPs are also reduced by an increased K^+^ conductance dependent on activation of D_1_ receptors.

## 2. MATERIALS AND METHODS

### 2.1. Tissue slices

Methods for obtaining whole cell current clamp recordings were similar to those described previously [13, 29, 33, 34]. Male Long-Evans rats between 4 and 6 weeks old were anesthetized with halothane, decapitated, and their brains rapidly removed and transferred into cold (4°C) artificial cerebrospinal fluid (ACSF) saturated with 95% O_2_ and 5% CO_2_ containing (in mM) 124 NaCl, 5 KCl, 1.25 NaH_2_PO_4_, 2 MgSO_4_, 2 CaCl_2_, 26 NaHCO_3_, and 10 dextrose (pH ≈7.3; 300–310 mOsm). All chemicals were obtained from Sigma-Aldrich, Mo, USA. Horizontal slices (300 *μ*m thick) were cut using a vibratome (WPI, Vibroslice, Fla, USA), and slices recovered for at least one hour at 22 to 24°C. Slices were transferred individually to a recording chamber and visualized using an upright microscope (Leica, Richmond Hill, Canada, DM-LFS) equipped with differential interference contrast optics, a 40x water immersion objective, and a near-infrared camera (COHU, Inc., Calif, USA). Submerged slices were superfused with oxygenated ACSF at a rate of 1.5 to 2.0 mL/min. Slices containing the lateral entorhinal cortex were taken from ventral sections about 1.9 to 3.4 mm above the interaural line [35]. Layer II was identified based on the presence of cell “islands” about 150 *μ*m from the cortical surface [36–39].

### 2.2. Stimulation and recording

Patch recording pipettes for whole cell recordings were prepared from borosilicate glass (1.0 mm OD, 4 to 8 MΩ) using a horizontal puller (P-97, Sutter Instr., Calif, USA) and were filled with a solution containing (in mM) 140 K-gluconate, 5 NaCl, 2 MgCl_2_, 10 HEPES, 0.5 EGTA, 2 ATP-Tris, and 0.4 GTP-Tris (pH adjusted to 7.24–7.32 with KOH; 270–280 mOsm). Pipettes were placed in contact with somata of layer II neurons, and gentle suction was applied under voltage clamp to form a tight seal (1–3 GΩ). Whole cell configuration was achieved by increased suction, and experiments began after cells stabilized (typically within 3 to 5 minutes after break-in). Current clamp recordings were obtained using an Axopatch 200B amplifier (Axon Instr., Calif, USA) and displayed on a digital oscilloscope (Gould 1604). Recordings were filtered at 10 kHz and digitized at 20 kHz (Axon Instr., Digidata 1322A) for storage on computer hard disk. Recordings were accepted if the series resistance was ≤25 MΩ (mean = 16.9 ± 0.9 MΩ) and if input resistance and resting potential were stable. A bipolar stimulating electrode made from two tungsten electrodes (FHC, 1.0 MΩ) was positioned to span layer I near the border with layer II approximately 0.2 to 0.6 mm rostral to the recording electrode. Synaptic responses were evoked with 0.1 millisecond constant current pulses delivered using a stimulus timer and isolation unit (WPI, Mass, USA, models A300 and A360). Stimulation intensity was adjusted to evoke responses approximately 75% of maximal (75 to 300 *μ*A).

All neurons (*n* = 118) included for analyses were identified as “fan” cells based on electrophysiological characteristics described previously [40, 41]. In comparison to stellate cells of the medial entorhinal cortex, fan cells show modest inward rectification during hyperpolarizing current steps, a small depolarizing afterpotential following single spikes, and do not show prominent theta-frequency membrane potential oscillations at subthreshold voltages [40–42].

### 2.3. Dopaminergic modulation of synaptic responses

The effects of dopamine on glutamate-mediated synaptic transmission in the lateral entorhinal cortex are largely uncharacterized. We therefore recorded both mixed and isolated components of excitatory postsynaptic potentials (EPSPs) evoked by stimulation of layer I before and after 5-minute bath-application of 1, 10, or 50 *μ*M dopamine. Results obtained using high concentrations of dopamine must be interpreted cautiously because of the possibility of nonspecific effects. However, dopamine degrades through oxidization within the slice preparation, and similar concentrations of dopamine have been used previously, and interpreted in light of the effects of specific antagonists, in reports examining the effects of dopamine on synaptic transmission in both the entorhinal [29–32] and prefrontal [23, 43] cortices. Responses were evoked once every 20 seconds, and the mean of 10 responses was obtained for analysis. Baseline responses were obtained at resting potential and, because dopamine usually hyperpolarizes fan cells, constant current was often required to return cells to the original membrane potential for recordings in the presence of dopamine. Sodium metabisulfite (50 *μ*M) was coapplied to slow the oxidation of dopamine [29, 31, 43], and ambient lighting was also reduced. Possible effects of sodium metabisulfite were assessed with a vehicle control group. Drugs were routinely stored at −20°C as concentrated stock solutions until needed, but dopamine HCl was dissolved just prior to bath application.

Paired-pulse tests were used to determine whether dopamine modulates EPSPs through a pre- or postsynaptic mechanism [13]. Pairs of stimulation pulses separated by an interval of 30 milliseconds were delivered before and after 5-minute bath-application of 1, 10, or 50 *μ*M dopamine. Stimulation intensity was reduced to evoke EPSPs approximately 50% of maximal, and ten responses were averaged for analyses. Paired-pulse facilitation was quantified by expressing the amplitude of the second response as a percentage of the first response.

Mechanisms mediating the suppression of EPSPs by high concentrations of dopamine were investigated by assessing the effects of 50 *μ*M dopamine on isolated components of synaptic responses. After baseline recordings in normal ACSF, AMPA receptor-mediated responses were isolated with bath application of 50 *μ*M 2-amino-5-phosphonovaleric acid (APV) and 25 *μ*M bicuculline methiodide, or NMDA receptor-mediated responses were isolated with 20 *μ*M 7-nitro-2,3-dioxo-1,4-dihydroquinoxaline-6-carbonitrile (CNQX) and 25 *μ*M bicuculline. GABA-mediated IPSPs were isolated with either 1 mM kynurenic acid or 20 *μ*M CNQX with 50 *μ*M APV. Isolated synaptic responses were recorded before and after 5-minute application of 50 *μ*M dopamine. Isolated AMPA receptor-mediated responses were also used to determine if dopamine suppresses EPSPs primarily through D_1_- or D_2_-like receptors. Baseline responses were recorded in the presence of either the D_1_ receptor antagonist SCH23390 (50 *μ*M) or the D_2_ receptor antagonist sulpiride (50 *μ*M) [29–31], and 50 *μ*M dopamine was then applied for 5 minutes. Sulpiride was prepared daily in a stock solution of 6% DMSO in ACSF titrated with 0.1 N HCl, and there was a final concentration of 0.1% DMSO with sulpiride.

The effects of dopamine on the intrinsic excitability of fan cells were assessed by monitoring responses to hyperpolarizing and depolarizing current steps. Changes in action potentials, afterhyperpolarizations, input resistance and inward rectification were examined before and after 5-minute bath application of 1, 10, or 50 *μ*M dopamine. The number of action potentials elicited in response to suprathreshold current injection can be used to characterize neuronal excitability [32], and we therefore determined the number of spikes fired in response to a single 500 millisecond-duration depolarizing current pulse from a constant holding potential (typically rest) using a pulse amplitude that elicited 3 to 5 action potentials [32]. Receptors that mediate the dopamine-induced change in input resistance were investigated using SCH23390 or sulpiride, and the ionic conductances involved were assessed using 0.5 *μ*M tetrodotoxin (TTX) or 30 mM tetraethylammonium (TEA). Blockers were preapplied for 5–10 minutes prior to coapplication of dopamine for 5 minutes.

### 2.4. Data analysis

Electrophysiological characteristics of fan cells and changes in synaptic responses were analyzed using the software program Clampfit 8.2 (Axon Instr., Calif, USA). The amplitudes of averaged EPSPs were measured relative to the prestimulus baseline, and paired-pulse facilitation was determined by expressing the amplitude of the second response as a proportion of the amplitude of the first response. Action potential amplitude was measured from resting potential, and action potential width and fast and medium afterhyperpolarizations were measured from threshold. Input resistance was calculated by measuring peak and steady-state voltage responses to −200 pA current steps (500 milliseconds), and inward rectification was quantified by expressing the peak input resistance as a proportion of the steady-state resistance (rectification ratio). All data were expressed as the mean ±SEM for plotting, and changes in response properties were assessed using paired samples *t*-tests or mixed design ANOVAs.

## 3. RESULTS

### 3.1. Electroresponsiveness of layer II fan cells

A total of 118 fan cells in layer II of the lateral entorhinal cortex were identified electrophysiologically and included for analysis, and the characteristics of these cells were similar to those reported previously [40, 41]. Fan cells had a mean resting membrane potential of −58.8 ± 0.6 mV, and a peak input resistance of 99.1 ± 2.1 MΩ. Most cells (108 of 118) demonstrated a small delayed inward rectification in response to hyperpolarizing current steps (rectification ratio: 1.10 ± 0.01). Action potentials (amplitude: 128.8 ± 0.7 mV, width: 4.1 ± 0.1 milliseconds, threshold: −44.1 ± 0.8 mV) were typically followed by fast and medium afterhyperpolarizations (−3.3 ± 0.3 mV and −5.8 ± 0.3 mV) with a small depolarizing afterpotential. Averaged EPSPs evoked by stimulation of layer I had a mean amplitude of 4.4 ± 0.2 mV. Continuous recordings of membrane potential were obtained in a subset of 28 cells to assess subthreshold membrane potential oscillations and, similar to findings of Tahvildari and Alonso [40], fan cells did not display prominent oscillations (data not shown).

### 3.2. Dopaminergic modulation of EPSPs

**Figure 1:**
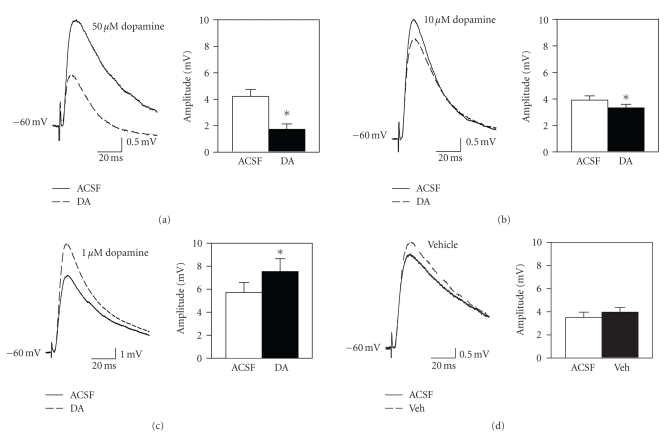
Dopamine has dose-dependent and bidirectional effects on the amplitude of mixed EPSPs in layer II fan cells. (a) Fifty *μ*M dopamine significantly reduces the amplitude of synaptic responses. Traces show averaged EPSPs before (ACSF) and after 5-minute bath application of dopamine (DA) in a representative cell. Group data indicate the mean amplitude of EPSPs before and after dopamine (*, *P* < .001). Bars indicate ± 1 SEM in this and subsequent figures, and * indicates *P* < .05 unless otherwise indicated. (b) A lower concentration of 10 *μ*M dopamine causes a smaller suppression of synaptic responses. (c) The low 1 *μ*M concentration of dopamine enhances the amplitude of synaptic responses (*, *P* < .01). (d) Bath application of vehicle (50 *μ*M sodium metabisulfite; Veh) does not significantly affect synaptic transmission.

We previously found concentration-dependent effects of dopamine on field EPSPs in layer II in vitro, in which 10 *μ*M dopamine facilitated fEPSPs and 50 to 100 *μ*M dopamine suppressed fEPSPs [29]. We obtained similar concentration-dependent effects in whole cell EPSPs recorded here before and after 5-minute bath application of dopamine. Application of 50 *μ*M dopamine resulted in a strong suppression of synaptic response to 38.5 ± 5.8% of baseline levels (see Figure 1(a); *t*_8_ = 7.75, *P* < .001; *n* = 9) that could be reversed by 15 minutes washout in normal ACSF (3 cells). We initially expected 10 *μ*M dopamine to facilitate EPSPs [29], but found that 10 *μ*M dopamine instead caused a small synaptic suppression (to 87.0 ± 5.8% of baseline; see Figure 1(b); *t*_15_ = 2.31, *P* < .05; *n* = 18). However, a lower concentration of 1 *μ*M dopamine significantly enhanced responses to 132.7 ± 4.4% of baseline levels (see Figure 1(c); *t*_6_ = 5.04, *P* < .01; *n* = 7). In our previous study using a gas-fluid interface chamber, a larger bath volume and slower flow-rate may have increased dopamine oxidation and reduced the effective concentration of dopamine at the slice, and this may account for why a higher applied concentration facilitated responses in that study [29]. Bath application of the antioxidant sodium metabisulfite alone had no significant effect on the amplitude of whole cell EPSPs (see Figure 1(d); *n* = 8).

**Figure 2:**
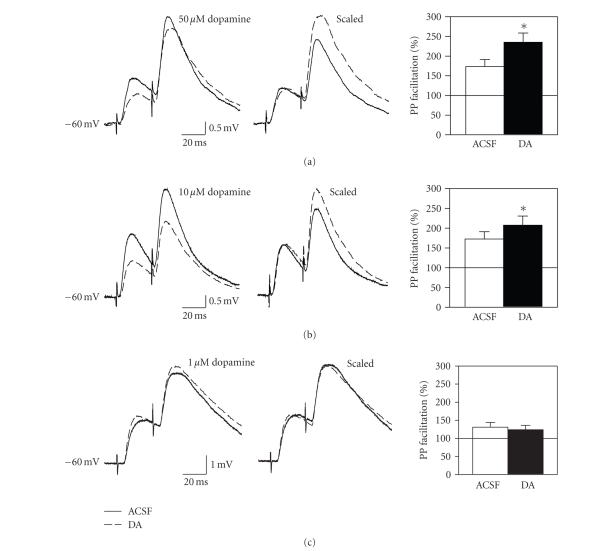
High concentrations of dopamine increase paired-pulse facilitation. (a) Pairs of stimulation pulses with a 30 millisecond interpulse interval were delivered before and after 5-minute bath application of 50 *μ*M dopamine. Averaged traces at left show responses recorded before (ACSF) and after (DA) dopamine from a representative cell. Note the suppression of the response to the first pulse and the large facilitation of the second response following dopamine (dotted line). Traces at right have been scaled to the amplitude of the first response in normal ACSF to aid comparison. Group data are shown on the right. (b) Paired-pulse facilitation was also enhanced by 10 *μ*M dopamine. (c) In contrast, the low concentration of 1 *μ*M dopamine does not affect paired-pulse ratio.

Paired-pulse tests were used to determine if synaptic suppression and facilitation effects were likely expressed pre- or postsynaptically. Pairs of pulses were delivered before and after 5-minute dopamine application, and a 30-millisecond interpulse interval was used that results in optimal paired-pulse facilitation [13, 44–46]. If EPSPs are reduced through a reduction in transmitter release, then a greater amount of transmitter should be available for release in response to the second stimulation pulse and paired-pulse facilitation should be enhanced [47–49]. Changes in EPSPs mediated by alterations in postsynaptic receptors, however, should not be associated with changes in paired-pulse ratio. High concentrations of dopamine that reduced EPSP amplitude were also found to enhance paired-pulse facilitation (see Figures 2(a), 2(b); *t*_13_ = 2.78, *P* < .05 for 10 *μ*M; *t*_8_ = 2.97, *P* < .05 for 50 *μ*M), suggesting that dopamine reduced EPSPs by suppressing glutamate release. In contrast, the low concentration of 1 *μ*M dopamine that facilitated EPSPs had no significant effect on paired pulse facilitation (see Figure 2(c)), suggesting that the facilitation of EPSPs was mediated primarily by an increased postsynaptic response to glutamate. The dopaminergic facilitation of the conditioning response was smaller during paired-pulse tests in which stimulus intensity was reduced to avoid spiking (see Figures 1(c) versus 2(c)) but a similar dopaminergic facilitation of fEPSPs with no effect on paired-pulse ratio has been observed in the entorhinal cortex in vivo [29].

### 3.3. Isolated synaptic responses

**Figure 3:**
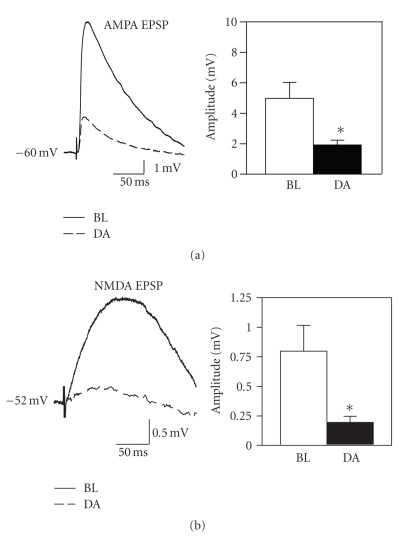
Dopamine suppresses the amplitude of both AMPA- and NMDA receptor-mediated components of EPSPs. (a) AMPA-mediated EPSPs recorded in the presence of APV and bicuculline were suppressed by 50 *μ*M dopamine. Averaged traces show EPSPs recorded before (BL) and after (DA) dopamine application, and group data are shown at right. (b) Isolated NMDA receptor-mediated EPSPs recorded in the presence of CNQX and bicuculline are also suppressed by a high concentration of dopamine. Group data show a consistent suppression of the small isolated NMDA response.

 The suppression of EPSPs by high concentrations of dopamine was examined more closely using pharmacologically isolated synaptic responses. Consistent with a suppression of glutamate release from presynaptic terminals, bath application of 50 *μ*M dopamine significantly attenuated both the isolated AMPA- and NMDA-mediated responses. The NMDA component was reduced to 26.0 ± 7.5% of baseline (see Figure 3(b); *t*_7_ = 3.32, *P* < .05; *n* = 8) and the AMPA component was reduced to 41.7 ± 5.6% of baseline (see Figure 3(a); *t*_5_ = 3.50, *P* < .05; *n* = 6).

**Figure 4:**
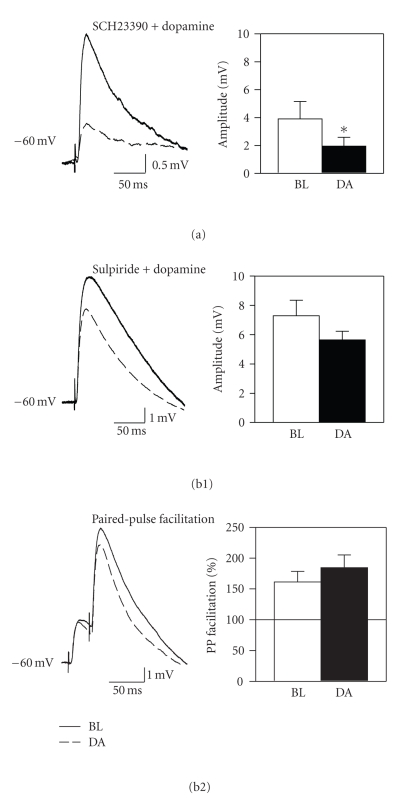
Dopamine suppresses isolated AMPA-mediated EPSPs via a D_2_ receptor-dependent mechanism. (a) Coapplication of the D_1_ receptor antagonist SCH23390 (50 *μ*M) did not prevent the dopamine-induced reduction in EPSP amplitude. (b) However, coapplication of the D_2_ receptor antagonist sulpiride (50 *μ*M) significantly attenuated the dopaminergic suppression of EPSPs. Sulpiride also prevented the enhancement of paired-pulse facilitation induced by dopamine (b2).

Dopamine receptor subtypes underlying the suppression of AMPA-mediated synaptic responses were investigated by applying 50 *μ*M dopamine in the presence of either the D_1_ receptor antagonist SCH23390 (50 *μ*M) or the D_2_ receptor antagonist sulpiride (50 *μ*M). Similar to previous reports that have used selective agonists in the medial [30, 31] and lateral [29] entorhinal cortex, application of either the D_1_ agonist SKF38393 (25 to 50 *μ*M; *n* = 9) or the D_2_ agonist quinpirole (20 to 40 *μ*M; *n* = 10) had no effect on EPSPs (data not shown), and we therefore used receptor blockers known to affect synaptic responses in the lateral entorhinal cortex [29]. Application of antagonists alone had no effect on EPSPs, and the D_1_ antagonist SCH23390 did not block the suppression of AMPA-mediated EPSPs (see Figure 4(a); *t*_4_ = 3.0, *P* < .05; *n* = 5), suggesting that D_1_ receptors do not mediate the suppression. However, blockade of D_2_ receptors with sulpiride significantly reduced the effects of dopamine on AMPA-mediated EPSPs. Coapplication of dopamine with sulpiride (*n* = 5) resulted in a nonsignificant suppression of synaptic responses, and the size of the suppression was significantly smaller than that observed with dopamine alone (79.8 ± 7.2% versus 41.7 ± 5.6% of baseline; F_1,9_ = 18.10, *P* < .001; see Figure 4(b1)). Sulpiride also prevented the enhancement of paired-pulse facilitation induced by 50 *μ*M dopamine (see Figure 4(b2)). Although this indicates that the dopaminergic suppression of EPSPs is largely dependent upon activation of D_2_-like receptors, the suppression of responses in the presence of sulpiride was close to statistical significance (*t*_4_ = 2.65, *P* = .06), suggesting that a non-D_2_ receptor-mediated mechanism mediates the residual suppression.

### 3.4. Dopaminergic suppression of IPSPs

**Figure 5:**
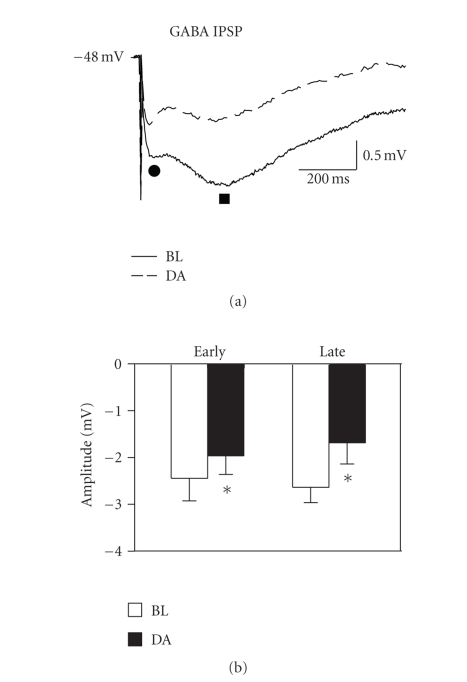
Dopamine suppresses both the fast and slow components of the mixed monosynaptic IPSP in fan cells. (a) GABA-mediated IPSPs were isolated pharmacologically with ionotropic glutamate receptor blockers and recorded at membrane potentials just below action potential threshold. Both the early (circle) and late (square) components of the biphasic IPSP were suppressed by 50 *μ*M dopamine (DA). (b) Group data reflect a significant suppression of both the early and late IPSPs.

Biphasic IPSPs were recorded from fan cells held near action potential threshold (−51 to −48 mV) and exposed to either 1 mM kynurenic acid or a combination of 50 *μ*M APV and 20 *μ*M CNQX to block ionotropic glutamate transmission. A concentration of 50 *μ*M dopamine suppressed both the early GABA_A_- and late GABA_B_-mediated components of the IPSP. The early IPSP was reduced to 84.5 ± 8.7% of baseline levels, and the late IPSP was reduced to 62.3 ± 11.1% of baseline levels (see Figure 5(b); early, *t*_8_ = 2.41, *P* < .05, *n* = 9; late, *t*_7_ = 2.46, *P* < .05, *n* = 8). The dopaminergic suppression of GABA synapses indicates that the reduction of EPSPs by dopamine is unlikely to be due to increased GABAergic inhibition of fan cells.

### 3.5. Modulation of intrinsic excitability

**Figure 6:**
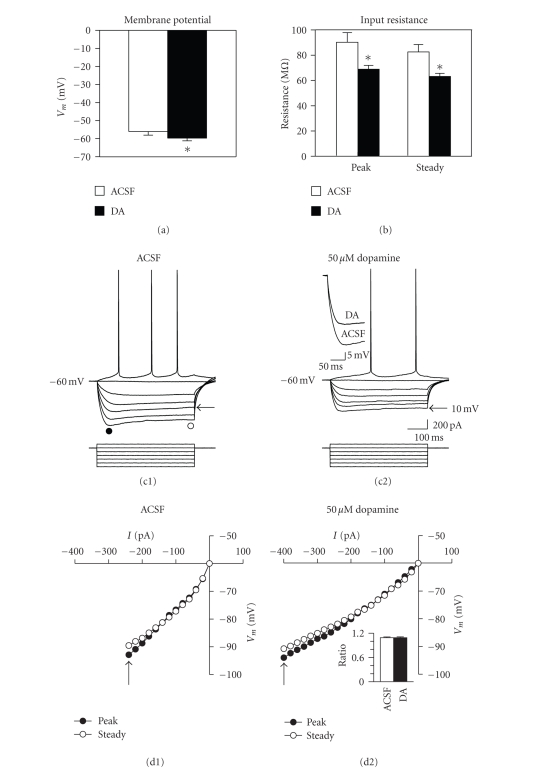
Dopamine hyperpolarizes membrane potential and reduces the input resistance of layer II fan cells. (a) Membrane potential was shifted to more hyperpolarized potentials by dopamine (*, *P* < .001). (b) Dopamine also reduced both peak and steady-state input resistance (*, *P* < .01). (c) Voltage responses to applied current steps before (c1) and after (c2) bath application of 50 *μ*M dopamine in a representative cell. Action potentials are truncated. Circles in (c1) indicate the latencies at which peak and steady-state input resistance were measured. Inset traces in (c2) compare the initial voltage deflection to a −200 pA current step before and after application of dopamine. Arrows indicate voltage responses before and after dopamine that were similar in amplitude and which allow comparison of the magnitude of the inward rectification. Note also the reduced input resistance across the entire range of hyperpolarizing current pulses. (d) Current-voltage plots show peak and steady-state responses to current steps of increasing size. Arrows indicate points at which a comparable degree of inward rectification was observed during hyperpolarization to similar voltages before and after dopamine application.

Bath application of dopamine also hyperpolarized resting membrane potential and reduced the input resistance of fan cells. Membrane potential was increased from −56.1 ± 2.0 to −59.7 ± 1.4 mV (see Figure 6(a); *t*_8_ = 4.73, *P* < .001; *n* = 9), and peak input resistance was reduced from 90.3 ± 7.6 to 68.9 ± 3.1 MΩ by 50 *μ*M dopamine (see Figure 6(b); *t*_7_ = 4.27, *P* < .01; *n* = 8). Similar changes in membrane potential and input resistance were observed for 10 *μ*M dopamine (not shown) and have also been reported following application of high concentrations of dopamine in whole-cell recordings from medial entorhinal cortex stellate cells [30]. Changes were not due to the vehicle, because control cells and cells exposed to 1 *μ*M dopamine did not show a drop in input resistance or hyperpolarization of membrane potential.

In layer V entorhinal cortex cells dopamine causes a reduction in excitability and a drop in input resistance through an increase in the hyperpolarization-activated current *I*_h_ [32], and changes in *I*_h_ were therefore assessed in layer II fan cells. However, dopamine did not significantly affect the amount of inward rectification, and the rectification ratio remained stable (see Figure 6(d); 1.09 ± 0.02 in ACSF and in 50 *μ*M dopamine, *t*_7_ = 0.00, *P* = 1.00).

**Figure 7:**
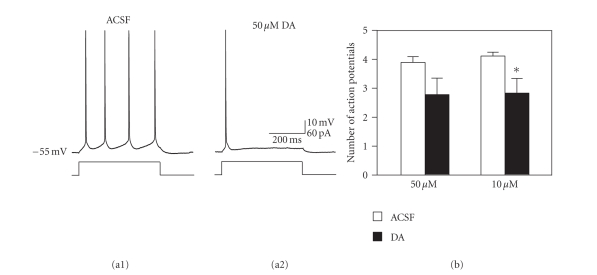
The number of action potentials elicited by positive current steps is reduced by dopamine. (a) Traces show action potentials generated in response to 500 milliseconds duration, 60 pA current steps before and after application of 50 *μ*M dopamine. The example shown reflects a particularly large reduction to only one action potential following application of dopamine. Action potentials are truncated. (b) Group data show a reduction in firing for both the 10 and 50 *μ*M conditions but only the reduction in the 10 *μ*M condition was significant.

Dopamine suppressed the excitability of fan cells, and application of 10 and 50 *μ*M dopamine reduced the number of action potentials evoked by brief 500 milliseconds depolarizing current pulses (see Figure 7). The number of spikes was reduced from 4.1 ± 0.1 to 2.8 ± 0.5 spikes by 10 *μ*M dopamine (see Figure 7(b); *t*_17_ = 2.54, *P* < .05; *n* = 18). A higher 50 *μ*M concentration of dopamine caused a similar reduction in the number of spikes (from 3.9 ± 0.2 to 2.8 ± 0.6) that was not statistically significant (*t*_8_ = 1.82, *P* = .11; *n* = 9). The reduction in spiking could result in part from reduced input resistance, but it was not due to membrane hyperpolarization because cells were tested at the same membrane potential both before and after dopamine application.

**Figure 8:**
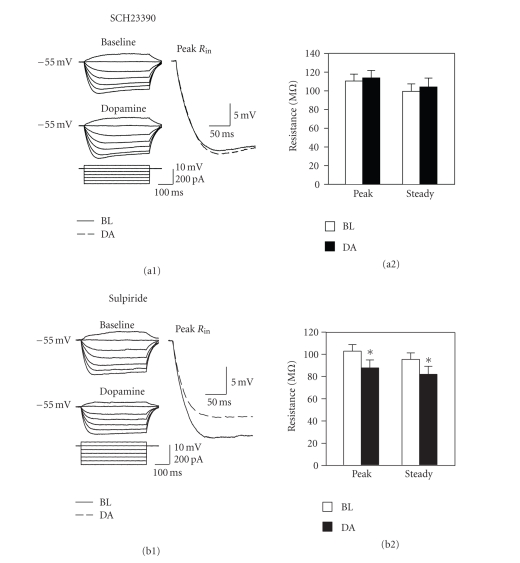
Blockade of D_1_, but not D_2_, receptors prevents the dopamine-induced reduction in input resistance. (a) Bath-application of the D_1_ receptor antagonist SCH23390 (50 *μ*M) prevented the reduction in input resistance induced by 50 *μ*M dopamine. Traces at left show voltage responses to a series of current steps during baseline recordings in SCH23390 and during subsequent dopamine application. Traces at right compare the initial voltage responses to −200 pA steps before and after dopamine application. Note that input resistance is unchanged when D_1_ receptors are blocked. (b) The D_2_ receptor blocker sulpiride (50 *μ*M) does not prevent changes in input resistance induced by dopamine (*, *P* < .001).

The drop in input resistance induced by 50 *μ*M dopamine was blocked by coapplication of the D_1_ receptor antagonist SCH23390 (and there was actually a very small but reliable *increase* in *R*_in_ in 4 of 5 cells; *t*_4_ = 2.60, *P* = .06; see Figure 8(a)). The drop in input resistance was not affected by coapplication of the D_2_ receptor antagonist sulpiride (*t*_4_ = 9.71, *P* < .001; *n* = 5; Figure 8(b)). The reduction in input resistance induced by dopamine is therefore dependent on activation of D_1_, but not D_2_, receptors.

**Figure 9:**
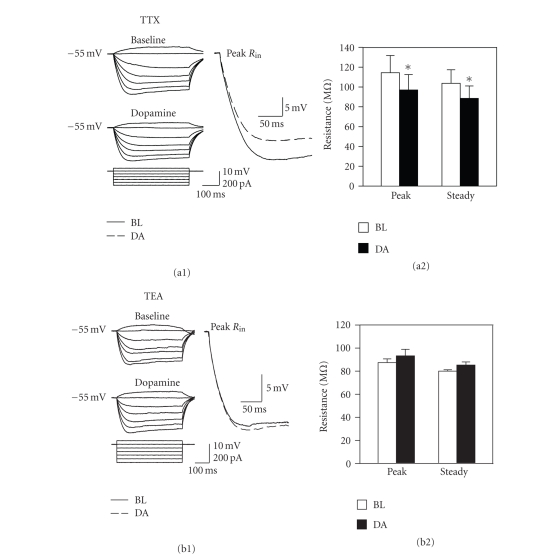
Blocking potassium channels prevent the dopamine-induced reduction in input resistance. (a) Blockade of Na^+^ channels with 0.5 *μ*M TTX does not prevent the reduction of peak or steady-state input resistance induced by 50 *μ*M dopamine (*, *P* < .01). Conventions are as in Figure 8. (b) In contrast, coapplication of the K^+^ channel blocker TEA (30 mM) prevented the dopamine-induced reduction in input resistance.

The conductances that mediated the reduced input resistance were investigated using blockers of Na^+^ and K^+^ channels. The Na^+^ channel blocker TTX was used to verify that reductions in input resistance were not due to an increase in action potential-dependent synaptic inputs to fan cells, or due to an altered Na^+^ conductance. Blockade of Na^+^ channels with TTX did not prevent the drop in input resistance induced by dopamine (see Figure 9(a); peak, *t*_4_ = 6.02, *P* < .01; steady-state, *t*_4_ = 8.21, *P* < .01; *n* = 5). It has been suggested that the reduced input resistance induced by dopamine in medial entorhinal cortex stellate cells might be mediated by an increased K^+^ conductance [30], and we therefore assessed the effects of dopamine on input resistance in the presence of the K^+^ channel blocker TEA (30 mM; *n* = 5). Coapplication of TEA blocked the reduction in input resistance induced by dopamine (see Figure 9(b)), indicating that the D_1_ receptor-dependent reduction in input resistance involves an increased K^+^ conductance. The increased K^+^ conductance is likely to contribute to the hyperpolarization of membrane potential induced by dopamine, and may also account for the reduced excitability of fan cells (see Figure 7). The reduced input resistance may also contribute to the dopamine-induced suppression of EPSPs; the D_2_ receptor blocker sulpiride did not fully prevent the suppression of AMPA-mediated EPSPs (see Figure 4(b1)), and the D_1_ receptor-mediated reduction in input resistance could contribute to part of the EPSP suppression.

## 4. DISCUSSION

We show here that dopamine has powerful suppressive effects on glutamate-mediated synaptic transmission in layer II fan cells of the lateral entorhinal cortex. Our findings suggest that the suppression of EPSPs involves the combined actions of a D_2_ receptor-mediated reduction in neurotransmitter release and a D_1_ receptor-mediated increase in a K^+^ conductance that reduces cellular input resistance. Previously, we found that field EPSPs were enhanced by low concentrations of dopamine in vitro, and by blocking dopamine reuptake in awake animals [29]. This suggested that moderate increases in dopamine release might facilitate synaptic responses in the entorhinal cortex, and enhance transmission of sensory information to the rest of the hippocampal formation. Here, we have replicated the synaptic facilitation with a low 1 *μ*M concentration of dopamine and have also shown that high concentrations of dopamine induce a strong and reversible suppression of intracellular EPSPs. Similar suppression effects have been observed in the medial entorhinal cortex [30, 31] and prefrontal cortex [22, 23, 50, 51] using comparable doses of dopamine.

### 4.1. Suppression of glutamate release

 The suppression of EPSPs by high concentrations of dopamine was found to be largely dependent on D_2_ receptors since coapplication of the D_2_ receptor antagonist sulpiride blocked most of the reduction. Dopamine also enhanced paired-pulse facilitation which suggests that the suppression of EPSPs resulted from a reduction in presynaptic glutamate release [47, 49]. The suppression of both AMPA- and NMDA-mediated components of the synaptic response is also consistent with reduced transmitter release. Although similar reductions in EPSPs have been shown in stellate cells of the medial entorhinal cortex, the suppression was dependent on D_1_, and not D_2_, receptor activation [30]. However, Stenkamp et al. (1998) showed a reduction in synaptic responses in layer III of the medial entorhinal cortex through activation of both D_1_ *and* D_2_ receptors, and results of paired-pulse tests in their study suggested that the suppression was also mediated by reduced glutamate release.

Dopamine has been shown to suppress AMPA-mediated synaptic responses in the prefrontal cortex through a D_1_ receptor-mediated suppression of transmitter release [22–24]. Strong activation of D_1_ receptors can also suppress synaptic responses through a retrograde signaling cascade. Weak D_1_ receptor activation can enhance NMDA responses, but stronger D_1_ receptor activation can lead to more intense NMDA receptor activation and the release of adenosine that suppresses transmitter release by acting on presynaptic A_1_ receptors that suppress voltage-gated Ca^2+^ channels [28, 52, 53]. In the striatum, activation of presynaptic D_2_ receptors suppresses *N*-type Ca^2+^ currents and inhibits acetylcholine release from striatal cholinergic interneurons [54]. D_2_ receptors have also been linked to a suppression of responses in the parabrachial nucleus [55], ventral tegmental area [56], and striatum [57, 58] via a D_2_-mediated reduction in glutamate release. A similar D_2_-mediated mechanism underlies the suppression of GABA release from striatal inhibitory cells onto cholinergic interneurons [59]. Similar mechanisms may mediate the dopaminergic suppression of glutamate release in the entorhinal cortex.

The dopaminergic suppression of EPSPs observed here cannot be explained by increased transmission at GABA synapses because we found that dopamine reduced monosynaptic GABA_A_ and GABA_B_ IPSPs. The suppression is also unlikely to be due to increased activation of feedback inhibition [60] because dopamine reduced both glutamatergic transmission and the number of spikes in fan cells (see Figure 7). The suppression of monosynaptic IPSPs that we observed may have resulted from a D_2_-mediated reduction in GABA release [59, 61] and reduced input resistance in fan cells could also have contributed. These possibilities are consistent with the parallel reductions observed in GABA_A_ and GABA_B_ IPSPs. Recordings of spontaneous and/or miniature IPSCs would be useful to determine the mechanisms of the reduced IPSPs.

### 4.2. Modulation of intrinsic excitability

In addition to the D_2_-mediated suppression of transmitter release, high concentrations of dopamine also appear to suppress synaptic transmission through a D_1_-receptor dependent mechanism. Sulpiride did not completely block the suppression of EPSPs (see Figure 4(b1)), and a D_1_ receptor-dependent activation of a TEA-sensitive K^+^ conductance appears to mediate the residual suppression via a reduction in input resistance. Blockade of synaptic transmission and voltage-gated Na^+^ channels with TTX did not prevent the drop in input resistance induced by dopamine indicating that it is not due to increased spontaneous synaptic drive or to an increased Na^+^ conductance. However, the broadly acting K^+^ channel blocker TEA prevented the drop in input resistance, indicating that dopamine activates a K^+^ conductance. The drop in input resistance was also prevented by blockade of D_1_, but not D_2_, receptors, indicating that dopamine activates K^+^ channels via D_1_ receptors. High concentrations of dopamine also hyperpolarize membrane potential and reduce input resistance in stellate cells of the medial entorhinal cortex, and it was also suggested that these changes might be mediated by an increased K^+^ conductance [30].

A large number of K^+^ conductances are affected by TEA, and it is therefore not clear which type(s) may be responsible for the drop in input resistance observed here. Background leak channels are insensitive to TEA [62] and are therefore not likely to contribute. Voltage-gated K^+^ currents are blocked by TEA, but dopamine in the prefrontal cortex tends to enhance neuronal excitability by *suppressing* these currents (see also [43, 63]). Several reports in CA1 pyramidal cells have found that dopamine hyperpolarizes membrane potential, reduces input resistance, and increases afterhyperpolarizations through a D_1_-receptor mediated increase in Ca^2+^-activated K^+^ currents ([64, 65], see also [66]), but others have found an increase in the excitability of CA1 neurons due to a *suppression* of Ca^2+^-activated K^+^ currents (see also [32, 67, 68]). Here, there was no clear increase in afterhyperpolarizations, suggesting that Ca^2+^-dependent K^+^ currents do not mediate the change in input resistance. Activation of D_1_ receptors can also have dose-dependent effects on activation of inward rectifying K^+^ currents (IRKCs). In the prefrontal cortex, D_1_ receptor activation typically *inhibits* IRKC by direct effects of cAMP on IRK channels, but strong activation can increase IRKC via phosphorylation of the channels through elevated levels of PKA [69]. This could explain why a significant reduction in input resistance was observed here only at the higher concentrations of dopamine. Clearly, however, further experiments will be required to determine the nature of the D_1_ receptor-dependent K^+^ conductance in fan cells.

 We observed a decrease in fan cell firing during depolarizing current steps after dopamine, and the reduced spiking may reflect the drop in cellular input resistance. A surprising finding was that while the D_1_ receptor antagonist SCH23390 prevented the dopamine-induced reduction in input resistance it did not completely eliminate the reduction in the number of spikes, suggesting that reduced input resistance cannot entirely account for the reduction in spiking, and that other mechanisms may also contribute. D_1_ receptor activation can *increase* spiking in prefrontal neurons by enhancing the persistent Na^+^ current (*I*_NaP_) and suppressing a slowly-inactivating K^+^ conductance [43, 70], but a suppression of spiking via a reduction in *I*_NaP_ has also been observed [71]. In layer V entorhinal cortex neurons, dopamine reduces input resistance and leads to a reduction of spiking though an increase in *I*_h_ [32]. Here, there was no apparent change in *I*_h_ in fan cells, and action potential threshold and afterhyperpolarizations were not affected, suggesting that the underlying currents were not modified. Dopaminergic effects on *I*_NaP_ were not directly assessed in the present study, and the drop in input resistance could mask possible reductions in depolarizing responses to current injection related to *I*_NaP_. However, in tests in which SCH23390 prevented a change in input resistance, we found no reduction in the response to +20 pA pulses. This argues against a D_1_-mediated reduction in *I*_NaP_, but it is still possible that dopamine may reduce spiking via a D_2_ receptor-mediated reduction in *I*_NaP_ [71].

## 5. CONCLUSIONS

 We have shown here that dopamine has concentration-dependent, bidirectional effects on glutamate-mediated synaptic transmission in principal cells of layer II of the lateral entorhinal cortex. The lateral entorhinal cortex receives a major input from the piriform cortex [5–7], and dopaminergic innervation of the superficial layers is likely to have a strong modulatory effect on olfactory processing. In the prefrontal cortex, moderate activation of dopaminergic inputs promotes working memory function, but excessive dopamine activation leads to a decrement in performance [20, 27]. In the entorhinal cortex, moderate increases in dopamine concentration may enhance the salience of olfactory representations carried to the lateral entorhinal cortex (see Figure 1(c); see also 29), but large increases in dopamine associated with drug effects or acute stress [27] may dampen synaptic inputs to the superficial layers and suppress working memory function [72–74] or induction of lasting synaptic plasticity [75]. The dopaminergic suppression of synaptic transmission in layer II is also likely to inhibit the propagation of sensory information to the rest of the hippocampal formation such that only strong and synchronous inputs to the entorhinal region may be sufficient to activate entorhinal projection neurons.
